# DW-Net: Dynamic Multi-Hierarchical Weighting Segmentation Network for Joint Segmentation of Retina Layers With Choroid Neovascularization

**DOI:** 10.3389/fnins.2021.797166

**Published:** 2021-12-24

**Authors:** Lianyu Wang, Meng Wang, Tingting Wang, Qingquan Meng, Yi Zhou, Yuanyuan Peng, Weifang Zhu, Zhongyue Chen, Xinjian Chen

**Affiliations:** ^1^School of Electronics and Information Engineering, Soochow University, Suzhou, China; ^2^State Key Laboratory of Radiation Medicine and Protection, Soochow University, Suzhou, China

**Keywords:** multi-target segmentation, choroid neovascularization, convolutional neural network, optical coherence tomography, medical image processing, attention mechanism

## Abstract

Choroid neovascularization (CNV) is one of the blinding factors. The early detection and quantitative measurement of CNV are crucial for the establishment of subsequent treatment. Recently, many deep learning-based methods have been proposed for CNV segmentation. However, CNV is difficult to be segmented due to the complex structure of the surrounding retina. In this paper, we propose a novel dynamic multi-hierarchical weighting segmentation network (DW-Net) for the simultaneous segmentation of retinal layers and CNV. Specifically, the proposed network is composed of a residual aggregation encoder path for the selection of informative feature, a multi-hierarchical weighting connection for the fusion of detailed information and abstract information, and a dynamic decoder path. Comprehensive experimental results show that our proposed DW-Net achieves better performance than other state-of-the-art methods.

## Introduction

**FIGURE 1:**
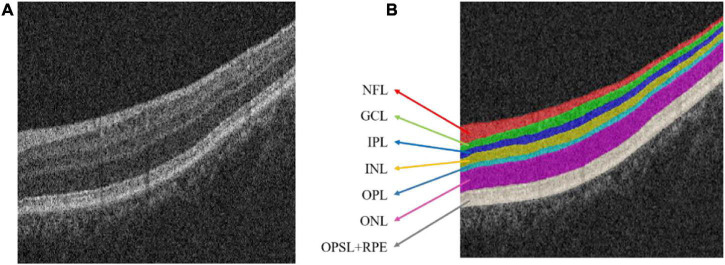
Optical coherence tomography (OCT) image of the normal retinal layer. **(A)** Original image. **(B)** Label. *NFL*, nerve fiber layer; *GCL*, ganglion cell layer; *IPL*, inner plexiform layer; *INL*, inner nuclear layer; OPL, outer plexiform layer; *ONL*, outer nuclear layer; *OPSL*, outer photoreceptor segment layer; *RPE*, retinal pigment epithelium.

The choroid is an important tissue of the human eye. It is a soft and smooth brown film located between the retina and the sclera (Hageman et al., 1995; Bressler, 2002). Optical coherence tomography (OCT) is a noninvasive, high-resolution biological imaging technology that can be used for *in vivo* measurement of fundus structures such as the retina, retinal nerve fiber layer, macula, and optic disc (Huang et al., 1991; Fercher et al., 1993). In OCT image, the normal retinal structure presents multiple interconnected retinal layers (Srinivasan et al., 2014; Zanet et al., 2019); from the inside to the outside are: the nerve fiber layer (NFL), Ganglion cell layer (GCL), inner plexiform layer (IPL), inner nuclear layer (INL), outer plexiform layer (OPL), outer nuclear layer (ONL), outer photoreceptor segment layer (OPSL), and retinal pigment epithelium (RPE). Figure 1 shows the OCT image with normal retinal layers.

**FIGURE 2:**
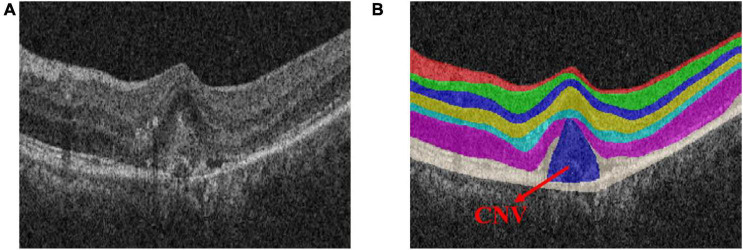
Optical coherence tomography (OCT) image of the normal retinal layer containing choroid neovascularization (CNV). **(A)** Original image. **(B)** Label.

Choroid neovascularization (CNV), also known as sub-retinal neovascularization, refers to the pathologically proliferating blood vessels that extend from the choroid to the sub-retinal pigment epithelium, the sub-retinal space, or a combination of the above (Lopez et al., 1991; Laud et al., 2006). Figure 2 shows the OCT image of the retina with CNV. Due to the high permeability of the vascular wall of neovascularization, it may lead to sub-retinal hemorrhage, lipid exudation, detachment of the retinal pigment epithelium and choroid, and the formation of fibrotic scars (Zhang et al., 2017). The main symptoms are visual loss, distortion of vision, and central or para-central dark spots, which eventually lead to blindness (Saxe et al., 1993; Grossniklaus and Green, 2004). Therefore, early detection and quantitative measurement of CNV are crucial for the establishment of subsequent treatment plans.

Medical-aided diagnosis segmentation algorithm based on computer vision can quickly obtain the shape, size, location, and optical density value, which can provide reliable and accurate quantitative information for the diagnosis and treatment of CNV (Chen et al., 2012, 2016; Gao et al., 2015). Therefore, the development of a reliable and automatic OCT-based CNV segmentation method requires further attention.

However, accurate segmentation of CNV still faces great challenges. Firstly, the structure of the retina is complex due to the multiple retinal layers it contains (Garvin et al., 2009; Roy et al., 2017). Secondly, with the existence of CNV or fluid, the adjacent retinal layers will deform greatly, resulting in a decrease in contrast (Shi et al., 2015). Thirdly, some CNVs are small objects that are hard to discriminate, resulting in performance degradation.

Therefore, focusing on these problems, we propose a new dynamic multi-hierarchical weighting segmentation network (DW-Net) for the joint segmentation of CNV and retinal layers in retinal OCT images. To alleviate the increase in the difficulty of CNV segmentation due to the complexity of the retinal layer structure, we developed a joint framework for the simultaneous segmentation of the retinal layers and CNV. To reduce the impact of partial deformation of the retinal layers and improve the segmentation performance on small CNVs, multiple multi-hierarchical connections are introduced in our proposed network, thus making full use of contextual information. Comprehensive experimental results suggest that our proposed DW-Net achieves superior performance in OCT-based segmentation of retinal layers with CNV compared with several state-of-the-art methods.

The major contributions of this paper can be summarized as follows. Firstly, we create an end-to-end deep learning framework for the simultaneous segmentation of the retinal layers and CNV. Secondly, we develop multiple multi-hierarchical connections to extract and fuse the features in a contextual-driven manner. Thirdly, we evaluate the proposed methods on OCT images of the retina, with experimental results suggesting the effectiveness of our methods.

The rest of the paper is organized as follows. We first briefly review related work in section “Related Work.” Then, we introduce the proposed dynamic multi-hierarchical weighting segmentation network (DW-Net) in section “Methods”. In section “Experiments and Results,” we present the experimental settings, experimental results, ablation study, and the materials used in this study. The ablation study and the limitations of our current work are shown in section “Discussion,” as well as possible future directions. Finally, we conclude this paper in section “Conclusion.”

## Related Work

In recent years, several automatic CNV segmentation methods of the retinal layers and CNV have been proposed. Lu et al. (2010) segmented the retinal blood vessel into multiple vascular and non-vascular slices, smoothed and filtered to refine the layer boundary. Song et al. (2013) further used arc-based graph representation, combined extensive prior information through paired energy terms, and calculated the maximum flow in low-order polynomial time. In the same year, Dufour et al. (2013) proposed a graph-based automatic multi-surface segmentation algorithm to add prior information from the learning model and further improved the accuracy of segmentation. Xu et al. (2013) used the Iowa reference algorithm to segment 10 retinal layers, followed by a combined graph search/graph cut method to segment pairs of adjacent retinal layers and any present fluid-associated abnormality detection region in 3D. Xi et al. (2017, 2018) developed a structure prior method based on sparse representation classification and local latent function to capture the global spatial structure and local similarity structure prior, which improved the segmentation robustness of CNVs of different sizes.

At present, deep neural networks have been widely used for the segmentation of retinal images and CNV. Su et al. (2020) proposed a differential amplification block to extract the contrast information of the foreground and background, which is integrated into the U-shaped convolutional neural network for CNV segmentation. Based on density cell-like P systems, Xue et al. (2018) proposed an automatic quantification method of the CNV total lesion area on outer retinal OCT angiograms to improve the accuracy of the segmentation boundaries. To simultaneously segment layers and neovascularization, Xiang et al. (2018) extracted well-designed features to find the coarse surfaces of different OCTs and introduced a constrained graph search algorithm to accurately detect retinal surfaces. Wang et al. (2020) trained two independent convolutional neural networks to classify the input scans according to the presence or absence of CNVs in a complementary manner, forming a powerful CNV description system.

## Methods

### Overview

**FIGURE 3:**
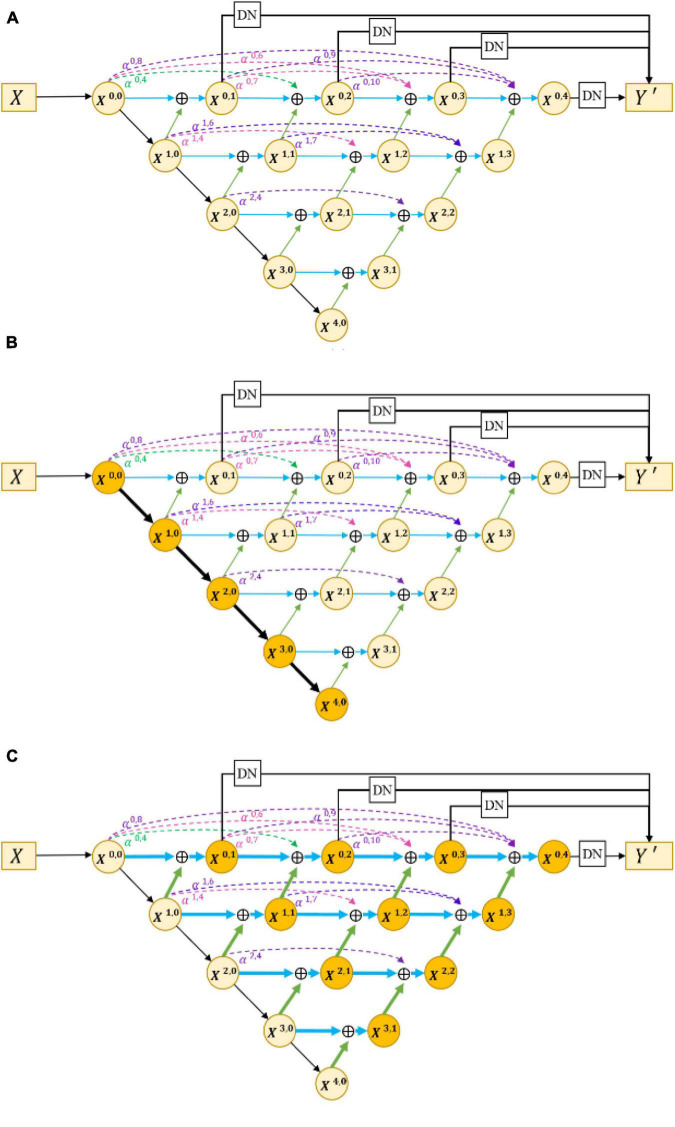
**(A)** Architecture of the proposed dynamic multi-hierarchical weighting segmentation network (DW-Net). The *dark yellow* part in **(B,C)** indicate the residual aggregation encoder path and the dynamic multi-hierarchical weighting connection, respectively.

The encoder–decoder structure (Ronneberger et al., 2015; Zhao et al., 2017; Feng et al., 2020) has been proven to be an efficient architecture for pixel-wise semantic segmentation among many deep learning-based methods; therefore, we propose a novel joint segmentation framework to solve the challenges in retinal CNV segmentation based on this. As shown in Figure 3A, the proposed DW-Net consists of three parts: residual aggregation encoder path, dynamic multi-hierarchical weighting connection, and dynamic decoder path.

### Residual Aggregation Encoder Path

In the conventional encoder path, encoders are composed of stacked convolutional layers and pooling layers, which are used to extract rich semantic information and global features layer by layer. However, continuous convolution and pooling will reduce the resolution of semantic features, which may lead to the loss of some small objects (such as small CNVs). To reduce the loss of resolution and enhance the selectivity of the feature encoder, we utilized the residual module as our encoder in this paper. By fusing the current feature maps with previous feature maps, the residual module can obtain informative feature maps that are more conducive to subsequent segmentation.

As shown in Figure 3B, the input data *X* ∈ ℝ^*H*×*W*×*C*^ is encoded by a convolutional layer and four encoders, as follows:


(1) $$\left\{ \begin{array}{c} X^{i,0}=\text{Conv}\,\left(X\right)\;i=0\;\\ X^{i,0}=R\,e\,s\,N\,e\,t\,\left(X^{i-1,0}\right)\,1\leq i\leq 4\, \end{array}\right.$$

where *H*, *W*, and *C* denote the height, width, and channels of the input data, respectively, and *X*^0,0^ represents the output of the first convolutional layer. *X*^*i*,0^(1 ≤ *i* ≤ 4) denotes the output feature maps of four encoders, with channel numbers of 64, 128, 256, and 512, respectively. To improve the feature extraction ability and save computing resources, we used the pre-trained model of layers 1–4 in ResNet18 (He et al., 2016) to initialize the parameters of the encoders.

### Dynamic Multi-Hierarchical Weighting Connection

Encoders of different hierarchies can extract features of different levels. The local features extracted by the low-level encoder are relatively simple and are more inclined to the basic components of images such as points, lines, and contours, while the high-level encoder is able to extract more complex features, such as abstract globe information. As for the semantic segmentation tasks, abstract global features can improve the overall positioning ability of the object, while fine local features can refine the edges of the segmented object.

**FIGURE 4:**
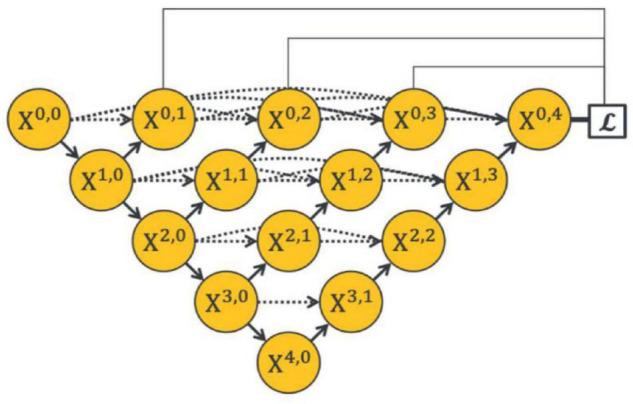
Architecture of the UNet++ by Zhou et al. (2018).

To make full use of the feature maps in multilevel encoders, as in Figure 4 (Zhou et al., 2018) developed UNet++. They concatenated the features of encoders in order layer by layer directly (gray dotted line in Figure 4), thus improving the performance of the segmentation network. However, the output feature map of the encoder usually contains interference information such as background and noise, which need to be selected and filtered. Also, the output features of each level have different contributions to the segmentation task; therefore, direct concatenation cannot highlight the importance of each part. In addition, concatenation in each hierarchy will greatly increase the parameters of the network, which may reduce the training and increase the risk of overfitting.

In response to the above problems, we proposed a dynamic multi-hierarchical weighting connection, which aims to take full advantage of the multi-scale extracted features that are conducive to segmentation in a contextual-driven manner and to filter irrelevant information. Figure 3C shows the structure of our proposed dynamic multi-hierarchical weighting connection, and its calculation process is as follows:


(2) $$X^{i,j}=\text{Conv}\,\left(X^{i,0}+D\,B\,\left(X^{x+1,j-1}\right)\right)\,0\leq i\leq 3,j=1$$

(3) Xi,j=Conv(∑k=0j-2αi,2⁢j+k*X+i,kXi,j-1 +DB(Xx+1,j-1))       0≤i≤4-j,2≤j≤4

where *i* and *j* denote the layer index and column index of the feature map *X*^*i*,*j*^, respectively, and *DB* represents a decode module composed of a 3 × 3 convolutional layer and an upsampling layer. α^*i*,2*j*+*k*^ is a learnable parameter, which is optimized through multiple iterations. To make full advantage of the known detailed information and abstract information at all hierarchies, we performed pixel addition on all higher-level feature maps and current-level feature maps according to their weight, thereby dynamically enhancing the segmentation ability of the current-level decoder.

### Dynamic Decoder Path

The dynamic decoder path contains four decoders, and the channels of the output feature map *X*^4−*i*,*i*^ (0 ≤ *i* ≤ 3) are 256, 128, 64, and 32, respectively. The decoder path is composed of stacked convolutional layers and upsampling layers, which aims to upsample the feature maps with strong semantic information from a high level and restore the spatial resolution layer by layer. Zhou et al. (2018), conducted pixel-wise averaging on the output feature map of the decoder path and output feature maps at the same hierarchy (the black straight line in the upper part of Figure 4), as follows:


(4) $$Y^{\prime }=\text{soft }\max\left(\frac{1}{4}\,\sum_{j=1}^{4}\text{Conv}\,\left(X^{0,j}\right)\right)$$

where *Conv* is a simple 1 × 1 convolutional layer for compressing the output feature channel. This strategy directly merges different feature maps without considering their depths. However, in convolutional neural networks, segmentation tasks are sensitive to the depth of the network; thus, a reasonable design of its depth will improve the performance (Simonyan and Zisserman, 2014). For this consideration, we modified the decoder path of UNet++ (Zhou et al., 2018) to extract more informative prediction results.


(5) $$Y^{\prime }=\text{soft }\max\left(\frac{1}{4}\,\sum_{j=1}^{4}D\,N\,\left(X^{0,j}\right)\right)$$

where *DN* is a dynamic fusion module consisting of a bilinear upsampling layer used to restore the input spatial resolution and two 1 × 1 convolutional layers followed by a normalization layer and a Relu nonlinear activation layer. Then, the 1 × 1 convolutional layer is applied for channel compression. Finally, we performed pixel-wise averaging on the output of *DN*, followed by a softmax layer. *Y*′ represents the predicted probability map.

### Loss Function

In the task of semantic segmentation of medical images, the pixel-by-pixel cross-entropy loss, ℒ_*CE*_, is a commonly used loss function that compares the predicted probability map with the gold standard (GT) in order according to the spatial position.


(6) $$Y^{\prime }=\text{soft }\max\left(\frac{1}{4}\,\sum_{j=1}^{4}D\,N\,\left(X^{0,j}\right)\right)$$

where *k* denotes the number of objects and *Y* represents the gold standard.

Dice loss, ℒ_*Dice*_, is another widely used loss function (Milletari et al., 2016) that aims to measure the overlap ratio of two samples, and its value ranges from 0 to 1.


(7) $${ℒ}_{D\,i\,c\,e}=1-\frac{1}{k}\,\sum_{c=0}^{k-1}\frac{2\,Y\,Y^{\prime }+\xi }{Y+Y^{\prime }+\xi }$$

where ξ is set to a very small constant to ensure that the divisor is not equal to 0. The final loss function we used is as follows:


(8) $$ℒ={ℒ}_{D\,i\,c\,e}+{ℒ}_{C\,E}$$

## Experiments and Results

### Dataset and Implementation

#### Dataset

To evaluate the effectiveness of the proposed method, we conducted comprehensive experiments. The dataset we used in the experiment was collected by the Joint Shantou International Eye Center of Shantou University and The Chinese University of Hong Kong. The acquisition process lasted 13 months, and 6,016 retinal OCT images from 47 three-dimensional retinal OCT volumes with CNV were completely acquired through the Zeiss canner. The size of the actual scanning area is 6 mm × 2 mm × 6 mm (*X* × *Y* × *Z*), and the number of voxels is 512 × 1,024 × 128. Pixel-level annotations of NFL, GCL, IPL, INL, OPL, ONL, OPSL+RPE, and CNV were provided by senior ophthalmologists.

#### Implementation Details

The implementation of our proposed DW-Net is based on the public platform Pytorch 1.8.0 with CUDA 11.0 parallel computing library and GeForce RTX 3090 GPU with 24-GB memory.

To save computing resources and increase network receptivity, each slice was resized to 512 × 512 by bilinear interpolation. We divided the 6,016 retinal OCT images into four groups, with the slice number as balanced as possible. Fourfold cross-validation was conducted on the divided dataset (1,664, 1,792, 1,280, and 1,280). The Adam optimizer with a learning rate of 1e-4 was adopted as our optimizer. The batch size and epoch were set to 4 and 100, respectively. For fair comparison, we used the same training strategy in all experiments.

#### Evaluation Metrics

Five metrics including dice similarity coefficients (DSCs), intersection-over-union (IoU), accuracy (Acc), sensitivity (Sen), and precision (Pre) (Garcia-Garcia et al., 2017) were used to fully and fairly evaluate the performance, where TN, TP, FN, and FP represent true negative, true positive, false negative, and false positive, respectively.


(9) $$\mathrm{DSC}=\frac{2TP}{2TP+\mathrm{FP}+\mathrm{FN}}$$

(10) $$\mathrm{IoU}=\frac{\mathrm{TP}}{\mathrm{TP}+\mathrm{FP}+\mathrm{FN}}$$

(11) $$\mathrm{Acc}=\frac{\mathrm{TP}+\mathrm{TN}}{\mathrm{TP}+\mathrm{FP}+\mathrm{TN}+\mathrm{FN}}$$

(12) $$\mathrm{Sen}=\frac{\mathrm{TP}}{\mathrm{TP}+\mathrm{FN}}$$

(13) $$\mathrm{Pr}e=\frac{\mathrm{TP}}{\mathrm{TP}+\mathrm{FP}}$$

### Results

We first compared our proposed DW-Net with other excellent convolutional neural network (CNN)-based methods, including UNet (Ronneberger et al., 2015), AttUNet (Oktay et al., 2018), CE-Net (Gu et al., 2019), Multi-ResUNet (Ibtehaz and Rahman, 2020), R2UNet (Alom et al., 2018), and DeepLab v3 (Chen et al., 2017). In addition, UNet++ (Zhou et al., 2018) was applied as our backbone. Tables 1, 2 show the mean joint segmentation results of the 7 retinal layers containing CNV and the joint segmentation results of CNV, respectively.

**TABLE 1:** Mean segmentation results (in percent) of the contrast experiments and ablation studies (mean ± SD).

Methods	DSC	IoU	Acc	Sen	Pre
UNet	94.01 ± 1.34	88.89 ± 2.27	99.23 ± 0.17	94.10 ± 1.30	94.03 ± 1.32
AttUNet	93.19 ± 0.38	87.48 ± 0.67	99.13 ± 0.07	93.27 ± 0.49	93.25 ± 0.33
CE-Net	94.98 ± 0.32	90.55 ± 0.57	99.36 ± 0.03	95.19 ± 0.17	94.84 ± 0.45
Multi-ResUNet	94.41 ± 0.31	89.57 ± 0.54	99.28 ± 0.04	94.42 ± 0.25	94.49 ± 0.38
R2UNet	88.19 ± 1.10	79.48 ± 1.61	98.49 ± 0.11	88.38 ± 1.29	88.67 ± 0.87
DeepLab v3	95.05 ± 0.10	90.69 ± 0.18	99.38 ± 0.01	95.26 ± 0.19	94.90 ± 0.10
Backbone	93.54 ± 0.39	88.07 ± 0.68	99.17 ± 0.08	93.69 ± 0.32	93.50 ± 0.51
DW-Net	**95.38** ± **0.22**	**91.26** ± **0.40**	**99.41** ± **0.02**	**95.44** ± **0.22**	**95.36** ± **0.23**

*Values in bold indicate the best performance. DSC, dice similarity coefficient; IoU, intersection-over-union; Acc, accuracy; Sen, sensitivity; Pre, precision.*

**TABLE 2:** Choroid neovascularization (CNV) segmentation results (in percent) of the contrast experiments and ablation studies (mean ± SD).

Methods	DSC	IoU	Acc	Sen	Pre
UNet	92.80 ± 2.17	87.15 ± 3.46	99.73 ± 0.08	93.12 ± 2.25	93.10 ± 1.72
AttUNet	91.27 ± 0.86	84.67 ± 1.31	99.68 ± 0.06	91.68 ± 1.73	91.75 ± 1.23
CE-Net	94.53 ± 0.92	90.00 ± 1.63	99.80 ± 0.04	**95.61** ± **0.95**	93.86 ± 2.17
Multi-ResUNet	93.70 ± 0.80	88.66 ± 1.26	99.77 ± 0.03	93.25 ± 0.88	94.72 ± 0.68
R2UNet	85.00 ± 3.48	75.64 ± 4.68	99.39 ± 0.02	89.52 ± 4.71	83.26 ± 3.17
DeepLab v3	93.74 ± 0.73	88.62 ± 1.23	99.77 ± 0.04	95.11 ± 0.76	92.77 ± 1.58
Backbone	92.51 ± 0.54	86.65 ± 0.85	99.72 ± 0.06	92.67 ± 0.94	92.99 ± 0.28
DW-Net	**94.84** ± **0.80**	**90.48** ± **1.38**	**99.81** ± **0.02**	95.13 ± 0.92	**94.81** ± **0.78**

*Values in bold indicate the best performance. DSC, dice similarity coefficient; IoU, intersection-over-union; Acc, accuracy; Sen, sensitivity; Pre, precision.*

From Table 1, it is worth noting that the proposed DW-Net achieves better performance than all of the above methods, with DSC, IoU, Acc, Sen, and Pre of 95.38, 91.26, 99.41, 95.44, and 95.36%, respectively. As for CNV, the performance of our proposed joint segmentation realized 2.52, 4.42, 0.09, 2.65, and 1.96% improvements in terms of DSC, IoU, Acc, Sen, and Pre, respectively, over the backbone, as shown in Table 2.

**FIGURE 5:**
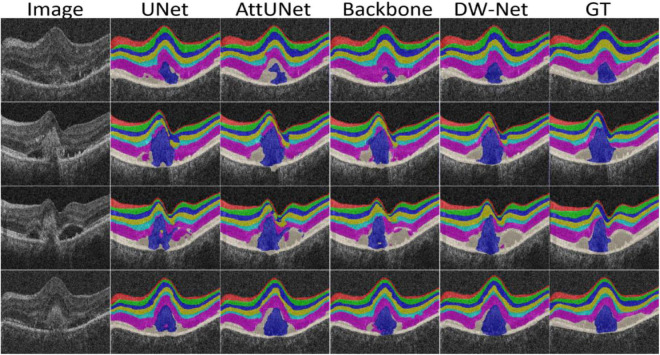
Visualization results of the different methods.

The performance of CE-Net (Gu et al., 2019) was comparable to that of the proposed DW-Net for CNV Sen, while being slightly lower in other metrics. In Figure 5, we plotted the visualization results of the different methods, where the red, green, dark blue, yellow, light blue, purple, white, and navy blue areas represent NFL, GCL, IPL, INL, OPL, ONL, OPSL+RPE, and CNV, respectively. It can be seen that our proposed DW-Net can accurately segment each retinal layer and CNV, which is closer to the GT compared with the other methods.

**FIGURE 6:**
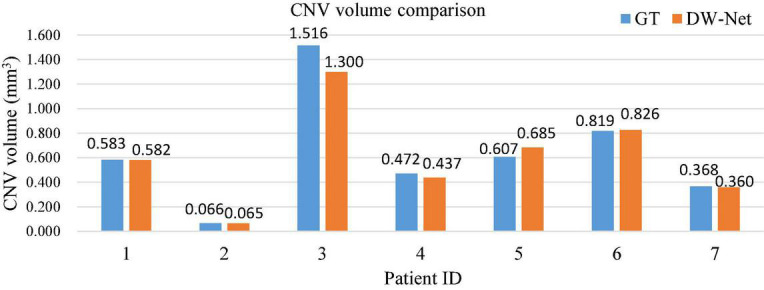
Histogram of choroid neovascularization (CNV) volume comparison.

Furthermore, we carried out a quantitative analysis of the experimental results. Figure 6 shows a histogram of the comparison between the size of the actual CNV and the segmented CNV using DW-Net, which are represented by blue and orange bars, respectively. It can be seen from the qualitative and quantitative results in the figure that the volume difference between the prediction of DW-Net and GT is generally small, which further proves the effectiveness and stability of the joint segmentation network and suggest promising clinical value and application prospects.

## Discussion

In this section, we first conduct a series of ablation experiments. Then, we study the contribution of the information on the retinal layers to the CNV segmentation task. Finally, we introduce the limitations of this work and possible future research directions.

### Ablation Experiments for Residual Aggregation Encoder Path

**FIGURE 7:**
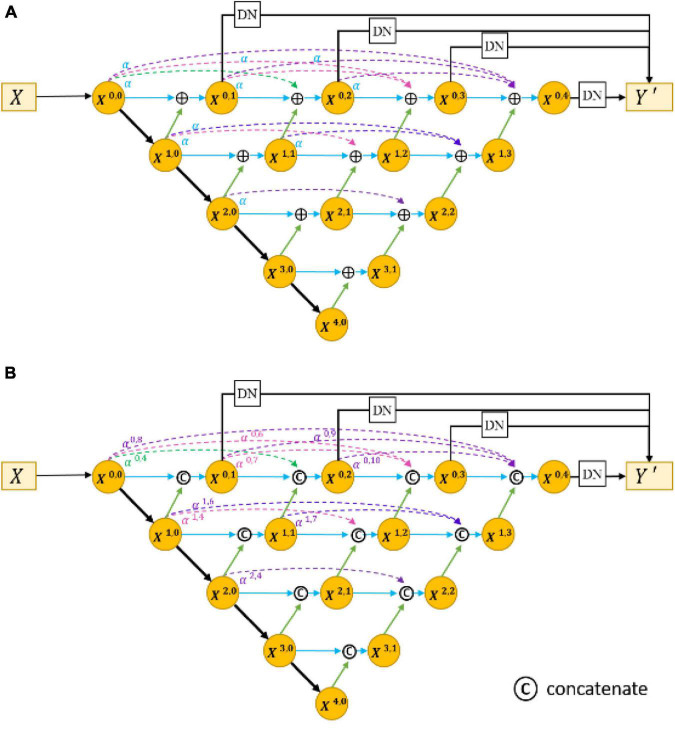
Architecture of Res18UNet++ **(A)** and AdaptiveUNet++ **(B)**.

To evaluate the effectiveness of the residual aggregation encoder path, we further compared the backbone with its counterparts (called Res18UNet++). Specifically, Res18UNet++ directly applies residual aggregation encoder path based on UNet++ (Zhou et al., 2018) and replaces concatenation by pixel addition, as shown in Figure 7A, where α is a constant that is fixed to 1. Table 3 reports the segmentation results.

**TABLE 3:** Ablation experiments (mean ± SD).

Methods	DSC	IoU	Acc	Sen	Pre
Backbone	92.51 ± 0.54	86.65 ± 0.85	99.72 ± 0.06	92.67 ± 0.94	92.99 ± 0.28
Res18UNet++	94.64 ± 0.60	90.21 ± 0.91	99.80 ± 0.03	94.65 ± 0.83	**95.02** ± **0.23**
AdaptiveUNet++	92.76 ± 0.60	87.06 ± 0.94	99.73 ± 0.05	92.96 ± 1.16	93.15 ± 0.75
DW-Net	**94.84** ± **0.80**	**90.48** ± **1.38**	**99.81** ± **0.02**	**95.13** ± **0.92**	94.81 ± 0.78

*Values in bold indicate the best performance. DSC, dice similarity coefficient; IoU, intersection-over-union; Acc, accuracy; Sen, sensitivity; Pre, precision.*

It can be seen that our proposed Res18UNet++ achieves better performance over the backbone on all metrics, which suggests that the residual aggregation encoder path can retain more effective features as possible to alleviate the resolution loss caused by network deepening.

### Ablation Experiments for Dynamic Multi-Hierarchical Weighting Connection

**FIGURE 8:**
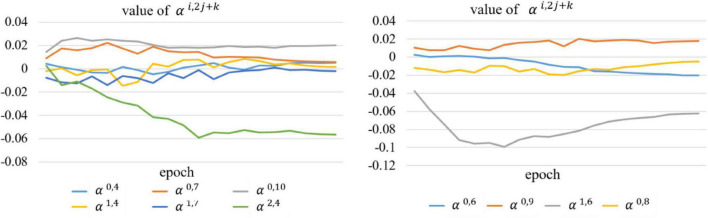
Value of the learnable parameter α^*i*,2*j*+*k*^ during training.

We also compared the backbone with another counterpart (called AdaptiveUNet++), as shown in Figure 7B. Here, α^*i*,2*j*+*k*^ is a learnable parameter that is multiplied with the output feature map of the corresponding encoder. Its value during the training process is shown in Figure 8.

We can conclude from Table 3 and Figure 8 that our proposed AdaptiveUNet++ enables the encoders to utilize multi-scale context information and filter irrelevant information. In addition, residual aggregation encoder path and dynamic multi-hierarchical weighting connection can influence and promote each other, thereby further improving the overall joint segmentation performance of the network, as shown in the results of DW-Net in Table 3.

### Ablation Experiments for Retinal Layer Information

All the experiments above were based on the assumption that the introduction of complex retinal layer information is conducive to improving the performance of CNV segmentation. Therefore, we performed joint segmentation of the retinal layers and CNV. In this section, we set out to verify the assumption.

#### Pre-processing

We considered CNV as the foreground, and the corresponding spatial label was set to 1, then the remaining area including the retinal layers was regarded as background, with the label of 0. Here, the joint segmentation was transformed into a foreground–background segmentation. A variant of DW-Net, named DW-Net-2, was applied for a single CNV segmentation, where the last layer of the network was modified to sigmoid function, and the number of output channels was set to 1. Table 4 shows the segmentation results of DW-Net-2 and DW-Net.

**TABLE 4:** Choroid neovascularization (CNV) segmentation experiments without retinal layers (mean ± SD).

Methods	DSC	IoU	Acc	Sen	Pre
DW-Net-2	90.06 ± 0.62	82.98 ± 0.90	99.63 ± 0.07	89.93 ± 0.50	91.52 ± 0.90
DW-Net	**94.84** ± **0.80**	**90.48** ± **1.38**	**99.81** ± **0.02**	**95.13** ± **0.92**	**94.81** ± **0.78**

*Values in bold indicate the best performance. DSC, dice similarity coefficient; IoU, intersection-over-union; Acc, accuracy; Sen, sensitivity; Pre, precision.*

It can be clearly seen that the performance of DW-Net is superior, which proves that the introduction of retinal layer information is conducive to distinguishing the features of background, retinal layers, and CNV, thereby improving the segmentation performance of CNV.

### Limitations and Future Work

The current work still has many limitations. Our proposed DW-Net contains many learnable parameters, which will increase the computational burden; therefore, further compression is needed in practical applications. The dataset used in our experiment needs further expansion, which is also one of our future works. We will conduct experiments on more datasets to verify the effectiveness and generalization of the proposed DW-Net.

## Conclusion

CNV segmentation is a fundamental task in medical image analysis. In this paper, we proposed a novel end-to-end dynamic multi-hierarchical weighting segmentation network (DW-Net) for the simultaneous segmentation of the retinal layers and CNV. Specifically, the proposed network is composed of a residual aggregation encoder path for the selection of informative feature, a multi-hierarchical weighting connection for the fusion of detailed information and abstract information, and a dynamic decoder path. Comprehensive experimental results show the effectiveness and stability of our proposed DW-Net and suggest promising clinical value and application prospects.

## Data Availability Statement

The datasets presented in this article are not readily available because constrained by ethics and patient privacy. Requests to access the datasets should be directed to corresponding author and LW, lywang12@126.com.

## Ethics Statement

The studies involving human participants were reviewed and approved by the Soochow University. Written informed consent for participation was not required for this study in accordance with the national legislation and the institutional requirements.

## Author Contributions

LW conceptualized and designed the study, wrote the first draft of the manuscript, and performed data analysis. MW, TW, QM, YZ, YP, ZC, and XC performed the experiments, collected and analyzed the data, and revised the manuscript. All authors contributed to the article and approved the submitted version.

## Conflict of Interest

The authors declare that the research was conducted in the absence of any commercial or financial relationships that could be construed as a potential conflict of interest.

## Publisher’s Note

All claims expressed in this article are solely those of the authors and do not necessarily represent those of their affiliated organizations, or those of the publisher, the editors and the reviewers. Any product that may be evaluated in this article, or claim that may be made by its manufacturer, is not guaranteed or endorsed by the publisher.
